# An Adverse Reaction in the Pediatric Sleep Laboratory

**DOI:** 10.1155/2016/9712579

**Published:** 2016-03-29

**Authors:** Diana Reppucci, Debra Medin, Suhail Al-Saleh, Mary Jane Smith, Jill Barter, Reshma Amin

**Affiliations:** ^1^Division of Respiratory Medicine, Department of Pediatrics, The Hospital for Sick Children, Toronto, ON, Canada M5G IX8; ^2^University of Toronto, Toronto, ON, Canada; ^3^Janeway Children's Health and Rehabilitation Centre, St. John's, NL, Canada A1B 3V6

## Abstract

We present a case of a 15-month-old boy with Cornelia de Lange Syndrome (NIPBL gene mutation). On a PSG, central sleep apnea (central apnea-hypopnea index of 19/hour) and nocturnal hypoventilation (transcutaneous CO_2_ > 50 mmHg for 53% of the night) were found. A positive pressure initiation study was aborted because the patient developed a serious adverse reaction. The differential diagnosis included a skin fragility condition versus an allergic contact dermatitis to the interface; this could be from the povidone-iodine solution used to clean the NiPPV interface or from the plastic of the interface itself. A skin biopsy was performed which was normal. The reaction was likely secondary to an allergic contact dermatitis from the povidone-iodine solution used to clean the NiPPV interface. The patient is currently tolerating NiPPV.

## 1. Case Presentation

**Figure 1:**
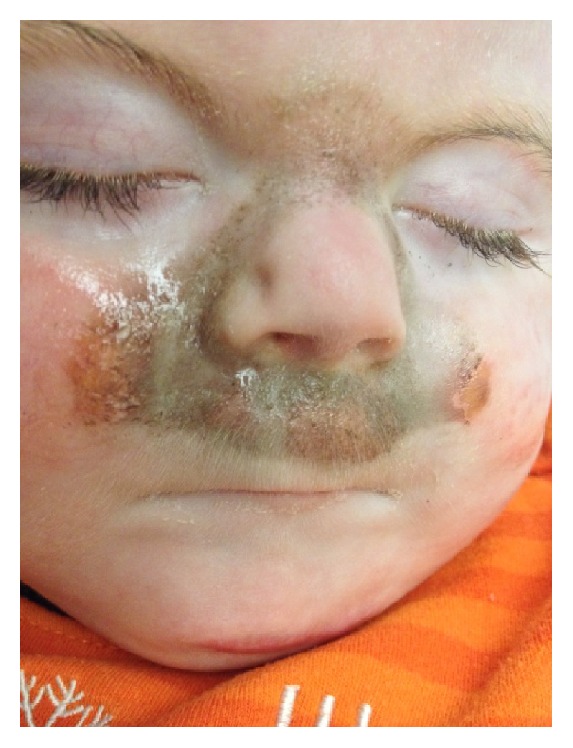
Severe, allergic contact dermatitis caused by the povidone-iodine cleaning solution for NiPPV interfaces within 4 hours of wearing the interface.

We present a case of a 15-month-old boy with Cornelia de Lange Syndrome (NIPBL gene mutation). He was referred for consultation due to recurrent, acute respiratory failure in the past 6 months. A polysomnogram (PSG) was performed because of clinically suspected obstructive sleep apnea (OSA). On the PSG, central sleep apnea (central apnea-hypopnea index of 19/hour) and nocturnal hypoventilation (transcutaneous CO_2_ > 50 mmHg for 53% of the night) were found. There were no obstructive respiratory events (obstructive apnea-hypopnea index of 0/hr). The patient was then brought back to the sleep laboratory the next night and noninvasive positive pressure ventilation (NiPPV) was initiated. The study was aborted 4 hours after the interface was placed due to a serious adverse reaction (see Figure 1).

## 2. Differential Diagnosis

The differential diagnosis included a skin fragility condition versus an allergic contact dermatitis to the interface; this could be from the povidone-iodine solution used to clean the NiPPV interface or from the plastic of the interface itself. A skin biopsy was performed. Immunohistochemical studies for epidermolysis bullosa were normal. Patch testing with povidone-iodine and its components was not done because of the risk of serious reaction. The skin lesions resolved within 6 weeks. In the interim, he was continued on supplemental oxygen. The family declined invasive ventilation via tracheostomy. A trial with the interface taped to the patient's arm was performed six months later using an identical NiPPV interface which had never been cleaned with povidone-iodine; there was no reaction. He is currently tolerating NiPPV.

## 3. Discussion

Povidone-iodine, a compound of iodine and povidone, with additives of glycerin, nonoxynol-9, disodium phosphate, citric acid, and polyoxyethylene nonylphenyl ether, is commonly used as a disinfectant and antiseptic agent for the treatment of contaminated wounds, the preoperative preparation of the skin and mucous membranes, and disinfection of equipment. Povidone-iodine has been reported to cause severe allergic contact dermatitis, generalized erythema multiforme-like eruption, and irritation of skin and mucous membranes [1–3]. Healthcare professionals initiating NiPPV with interfaces cleaned with povidone-iodine should be aware of this potential reaction. Furthermore, there should be an ongoing assessment for the potential development of an allergic contact dermatitis to such NiPPV interfaces during the night in the sleep laboratory or on the inpatient units.
